# The role of small GTPases in Alzheimer’s disease tau pathologies

**DOI:** 10.3389/fncel.2025.1650400

**Published:** 2025-09-25

**Authors:** Peter Hoegy, Yan-Hua Chen, Qun Lu

**Affiliations:** 1 Department of Chemistry and Biochemistry, University of South Carolina, Columbia, SC, United States; 2 Center for Neurotherapeutics, University of South Carolina, Columbia, SC, United States

**Keywords:** Alzheimer’s disease, small GTPases, microtubule-associated protein tau, hyperphosphorylation, aggregation, propagation, clearance, neurofibrillary tangles

## Abstract

Microtubule-associated protein (MAP) tau stabilizes neuronal microtubules in axonal transport and contributes to healthy synapses. In Alzheimer’s disease (AD), tau proteins become hyperphosphorylated, reduce microtubule binding, and aggregate into paired helical filaments (PHFs) in neurofibrillary tangles (NFTs). Although the steps of this dysregulation of tau are well established, the mechanisms by which each step is regulated remain incompletely understood. Misfolded protein aggregates, such as amyloid *β*-peptides (Aβ), are degraded by autophagy and lysosomal pathways, in which small GTPases play essential roles. However, how tau aggregates and spreads from nerve cells and whether small GTPases similarly play pivotal roles are not as clear. Here we review the recent evidence to propose that small GTPases are important in tau protein posttranslational phosphorylation, aggregation, and clearance. As such, small GTPases may prove to be important therapeutic targets that can reduce the AD tau burden.

## 1 Physiological roles of amyloid precursor proteins (APPs) and microtubule-associated protein (MAP) tau

Intracellular neurofibrillary tangles (NFTs) and extracellular amyloid plaques (or senile plaques, SPs) are the two most prominent hallmark pathologies of Alzheimer’s disease (AD) (Plascencia-Villa and Perry, 2022). SP consists of a core structure of Aβs, which are derived from the much larger amyloid precursor protein (APP) (Zheng and Koo, 2011). While APP plays important roles in normal physiology (Zheng et al., 1995), the overburden of Aβ deposition in brain tissue elicits a cascade of events that are harmful for the brain (Jagust, 2016).

On the other hand, microtubule-associated protein (MAP) tau is the major component of NFTs (Grundke-Iqbal et al., 1986; Ihara et al., 1986; Kosik et al., 1986; Wood et al., 1986). Tau proteins are the integral component of neuronal microtubules where proteins synthesized in the neuronal perikaryon are transported using microtubules as a “railroad” track to the distant synapses. Under physiological conditions, tau proteins localize selectively to axons and stabilize axonal microtubules, whereas other MAPs, such as MAP2, are localized predominantly to the dendrites (Binder et al., 1986). In addition to the role of tau protein as a stabilizer of microtubules, tau has demonstrated involvement in a collection of other cellular processes. Several studies have demonstrated tau interactions with NMDA receptors can regulate NMDA receptor activity, indicating a possible role in synaptic plasticity (Sinsky et al., 2021; Mondragón-Rodríguez et al., 2012). Furthermore, tau has exhibited the ability to bind to nucleic acids to prevent oxidative damage, as well as regulate axonal myelination (Kent et al., 2020; Sinsky et al., 2021).

While Aβ peptides are linked to AD pathologies, it is now postulated that they may also play important roles in protecting brain functions. For example, studies suggest that Aβ may be a response to pathogen-induced neuroinflammation (Wozniak et al., 2009; Eimer et al., 2018; Itzhaki et al., 2016; Jorfi et al., 2023). Similarly, the hyperphosphorylation of tau proteins is integral in AD pathologies but is increased during normal development and is involved in regulating neurite outgrowth (Stoothoff and Johnson, 2005). There is also evidence that tau displays a transient protective effect against Aβ-induced cognitive impairments in animal models (Emmerson et al., 2024).

## 2 AD tau proteins and their roles in disease progression

In AD, earlier investigations discovered that NFTs consist of, almost exclusively, hyperphosphorylated tau proteins (Grundke-Iqbal et al., 1986; Wood et al., 1986; Ihara et al., 1986). The follow up studies showed that AD brain lysates enriched with tau proteins displayed a reduced taxol-stabilized microtubule binding capability (Nieto et al., 1991; Bramblett et al., 1992). Lu and Wood (1993) provided direct evidence in a binary interaction study that tau proteins isolated from AD brain are defective at promoting tubulin assembly. The observation was further supported by Yoshida and Ihara (1993) that tau in PHF is almost assembly-incompetent. These studies thus validated the hypothesis that AD tau is compromised in its functions to nucleate, bundle, and stabilize microtubules.

On the other hand, it is hypothesized that hyperphosphorylated AD tau proteins are more readily detached from microtubules and form aggregates to spread from one brain region to another (Miao et al., 2019; Wegmann et al., 2021). The tau aggregates are likely to induce the increased detachment of microtubule-bound tau proteins and thus further destabilize axonal microtubule systems, leading to synaptic loss and dementia. However, the molecular mechanisms by which the tau aggregates form and spread are not completely understood. Recent studies suggest that in addition to abnormal phosphorylation, other factors like polyanions can influence tau aggregation (Fichou et al., 2018). RNA, the most potent polyanion trigger of tau aggregation *in vitro* (Kampers et al., 1996), appears to be the most abundant in the neuronal cytoplasm (McMillan et al., 2023). It is likely that a disrupted nuclear-to-cytoplasmic transport can lead to nuclear RNAs mis-localizing to the cytoplasm where they bind to tau proteins to promote tau aggregation. Studies have shown that tau aggregates released from the neurons can spread to other neurons by macropinocytosis (Wu et al., 2013; Wu et al., 2016; Evans et al., 2018). In this regard, defective intracellular trafficking and vesicular transport are also a likely culprit of AD tau pathogenesis.

## 3 Small GTPases: their physiological roles in cell signaling

It is important to note that the defective intracellular transport underlies the formation of many neuropathological inclusions such as Aβ and TDP-43 (Chou et al., 2018; Zhuravleva et al., 2021). The potential links between mis-localized RNAs and tau aggregation suggest that this crucial dysregulation occurs at intracellular trafficking and transport of cellular organelles and proteins, where small GTPases play a pivotal role.

**Figure 1:**
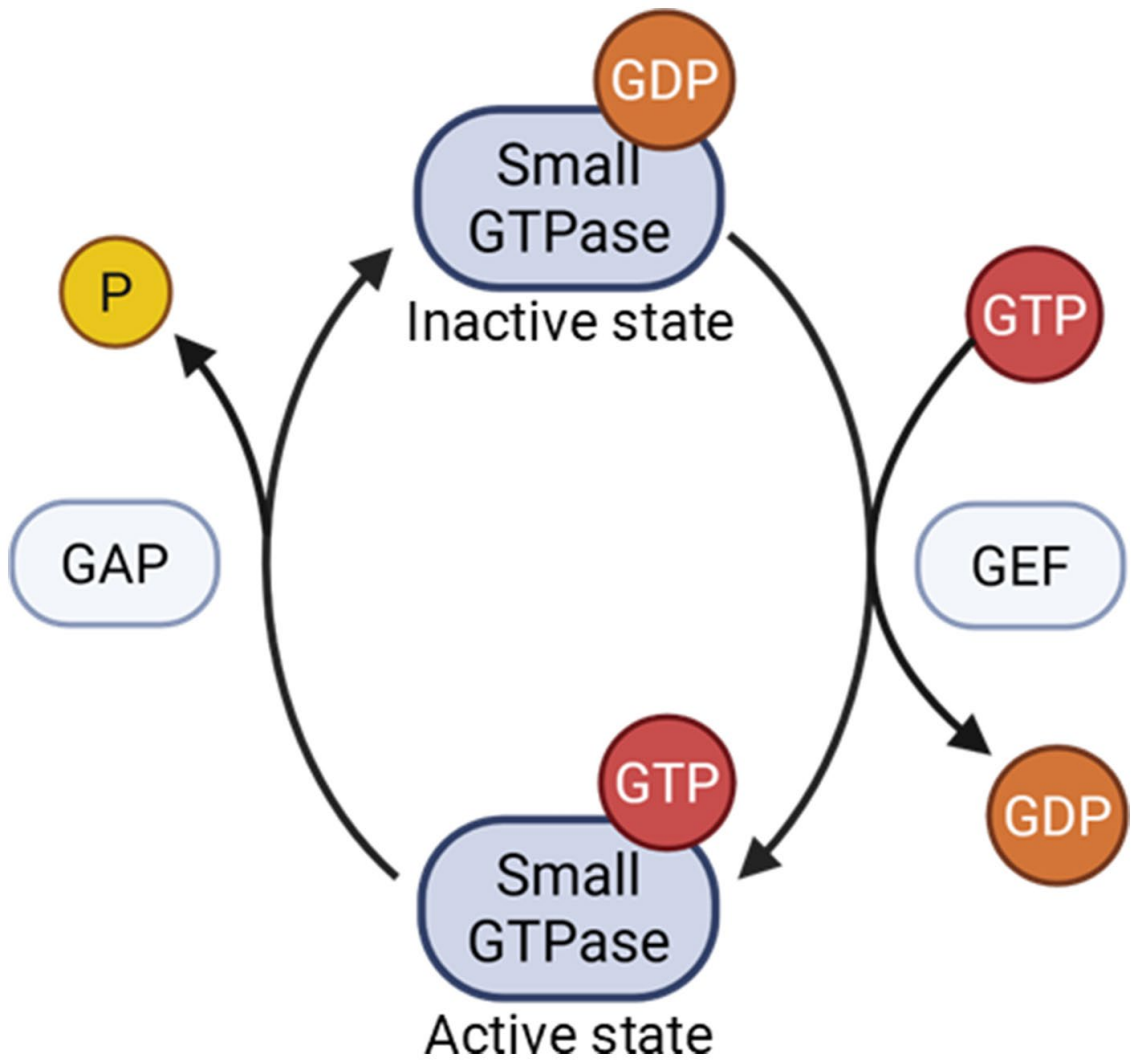
Small GTPases cycle between a GDP-bound/inactive state through GAP activity and a GTP-bound/active state through GEF activity.

Small GTPases of Ras superfamily are 21 kDa enzymes that bind and hydrolyze guanosine triphosphate (GTP). They are molecular switches that activate and regulate downstream signaling pathways (Dautt-Castro et al., 2024). Small GTPases have two states: an active GTP-bound state and an inactive GDP-bound state (Figure 1). Guanine nucleotide exchange factors (GEFs) can remove GDP from a small GTPase in exchange for GTP, activating the complex. GTPase activating proteins (GAPs) inactivate GTP-bound small GTPases by accelerating the hydrolysis of the bound GTP to GDP. GDP dissociation inhibitors, or GDI, bind to small GTPases and prevent the dissociation of GDP that keeps the enzymes in an inactive state (Aguilar et al., 2017). GDI proteins also prevent small GTPases from interacting with organelle membranes to activate further signaling.

**Figure 2:**
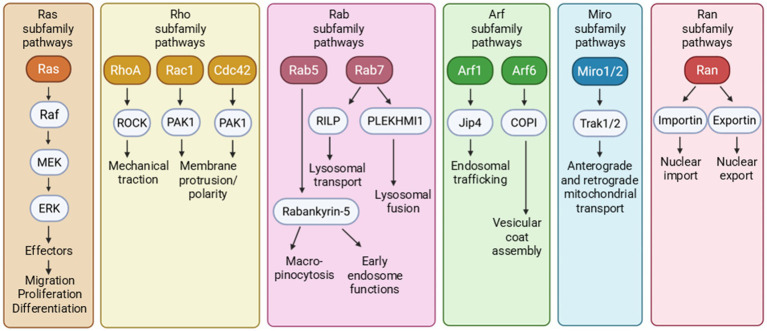
The small GTPases of Ras superfamily encompass multiple small GTPase subfamilies that affect numerous downstream cellular functions.

Members of the Ras superfamily regulate signaling pathways involved in many cellular processes. The most prominent small GTPase subfamilies include Ras, Ran, Rho, Rab, Arf, and Miro, which control proliferation, nuclear transport, cytoskeletal regulation/dynamics, membrane trafficking, vesicular transport, and mitochondrial transport, respectively (Kay et al., 2018; Jeong et al., 2022; Arrazola Sastre et al., 2020) (Figure 2). The Ras superfamily also extends to more than 170 small GTPases that include GEM and RRAG involved in autophagy and lysosomal functions (Colicelli, 2004). While many of these cellular functions are dysregulated in AD and related dementias (ADRDs), impaired intracellular protein transport and trafficking are among the earliest neuropathogenic events, including defective protein processing and phosphorylation, as well as misfolding and aggregation (Ferreira et al., 1997; Cataldo et al., 2000; Hunter et al., 2013; Wegmann et al., 2021). As we review the evidence, we hypothesize that small GTPases are important in tau protein posttranslational phosphorylation and distribution, aggregation and propagation, and clearance.

## 4 The roles of small GTPases in tau hyperphosphorylation

**Figure 3:**
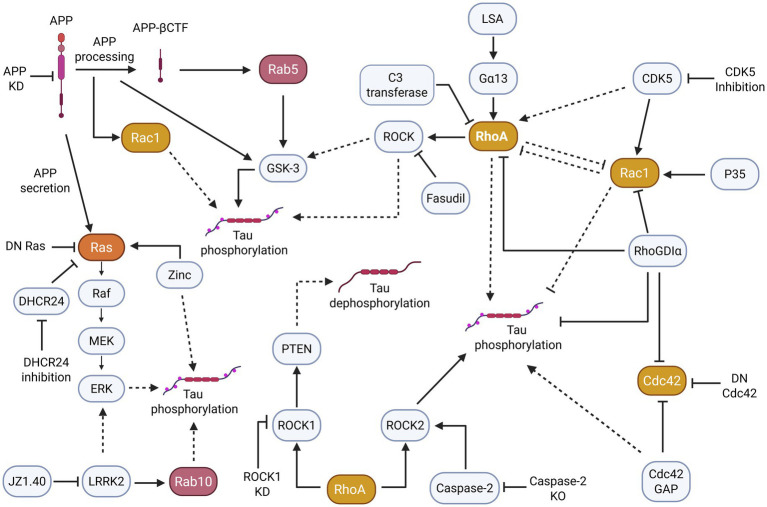
Small GTPase pathways, particularly those of the Ras, Rho, and Rab subfamilies appear to indirectly and directly induce changes in tau phosphorylation. More specifically, the dysregulation/overactivation of small GTPases and their pathways often contribute to tau hyperphosphorylation. Solid arrowheads indicate observed or reported connections that activate/upregulate a protein, signaling molecule, or cellular process; dotted arrowheads indicate unknown or hypothetical/indirect connections that activate/upregulate a protein, signaling molecule, or cellular process. Solid flathead arrows indicate observed or reported connections that inhibit/downregulate a protein, signaling molecule, or cellular process; dotted flathead arrows indicate unknown or hypothetical/indirect connections that inhibit/downregulate a protein, signaling molecule, or cellular process.

The hyperphosphorylation of tau is an important step in AD tau pathogenesis. Evidence suggests that small GTPases and related signaling networks play a role in tau hyperphosphorylation (Figure 3).

In AD disease models, the Rab, Rho, and Ras small GTPase subfamilies are implicated in tau phosphorylation through APP exposure. For instance, one study demonstrated that Rab5 was overactivated in AD and was typically mediated by APP-*β* secretase cleaved C-terminal fragment (APP-βCTF) (Pensalfini et al., 2020). However, Rab5 could also be activated independently of APP-βCTF in the PA-Rab5 mouse model, causing increased glycogen synthase kinase-3β (GSK-3β) activity to promote tau hyperphosphorylation. A classical member of the Rho GTPase subfamily, Rac1 additionally exhibited abnormal activation, as seen in the hippocampus of 6-week-old 3xTg-AD mouse model (Borin et al., 2018). The *in vitro* investigation of Rac1-specific signaling demonstrated that Rac1 activation induces tau hyperphosphorylation at residue pT181 through the increased processing of APP.

Other studies demonstrate that with elevated APP exposure, Ras signaling is similarly increased, causing various downstream effects. In PC12 cells, secreted APP stimulates MAPK or ERK (extracellular signal-regulated kinase), an effector of the Ras pathway (Greenberg et al., 1994). Further, a PC12 dominant inhibitory Ras cell line prevented secreted APP from stimulating MAPK/ERK, impeding tau phosphorylation, while the opposite effect was observed in cell lines without Ras inhibition. Likewise, APP knockdown (KD) in B103 rat neuroblastoma cells inhibited Ras–ERK activity. Finally, primary rat neurons treated with Aβ produced selective phosphorylation of adult tau isoforms (Ferreira et al., 1997) and increased Ras–ERK signaling and GSK-3 activation along with phosphorylated tau (Kirouac et al., 2017).

Interestingly, in a mouse model of Parkinson’s disease (PD), overexpressed *α*-synuclein in E18 cortical neurons induced a notable increase in the activation of small GTPases Rab5 and Rab7 (Fang et al., 2017). This increased Rab subfamily activation did not coincide with abnormal tau phosphorylation, suggesting that alternative pathways may induce tau hyperphosphorylation in PD tauopathies.

In addition to APP playing a role in small GTPase-induced tau hyperphosphorylation, the related GSK-3 pathway is involved in modulating tau phosphorylation. Namely, the Rab and Ras subfamilies have been implicated in regulating the GSK-3 pathway that leads to an increase in phosphorylated tau in murine models (Kirouac et al., 2017; Pensalfini et al., 2020). Along with these subfamilies, Rho subfamily small GTPases appear to have a connection with GSK-3 and tau hyperphosphorylation as well. This relation was exemplified in rat cerebellar granule neurons, in which lysophosphatidic acid (LPS) induced G protein subunit Gα13 to interact with RhoA (Sayas et al., 2002). This interaction activated GSK-3β to induce tau hyperphosphorylation and could be blocked by RhoA inhibition with C3 transferase.

Related to the GSK-3 signaling pathways are the MAPK/ERK signaling pathways associated with Ras, which may likewise induce tau hyperphosphorylation. MAPK/ERK is a downstream effector of the Ras/Raf cascade which can regulate and be regulated by GSK-3 (Ding et al., 2005; Wang et al., 2006). Expanding upon the studies described above, increased MAPK/ERK activity can led to increased tau phosphorylation (Greenberg et al., 1994; Kirouac et al., 2017). In fact, MAPK/ERK can convert wildtype tau to hyperphosphorylated tau and reduce its ability to promote microtubule assembly. Additionally, microinjected MAPK/ERK can result in AD-like tau phosphorylation at serine 396/404 (PHF-1) residues in cultured hippocampal neurons (Lu et al., 1993).

Additional literature supports this evidence. In one study, treating human wild-type tau-expressing SH-SY5Y cells with zinc increased the phosphorylation of serine 214 in tau, whereas suppression of the Ras–Raf-MAPK pathway inhibited this phosphorylation (Kim et al., 2011). Conversely, synthetase 3β-hydroxysterol-Δ24 reductase (DHCR24) KD in C8D1A astrocytes activated the rafts/caveolae-dependent Ras–MEK–ERK signaling pathway to induce tau hyperphosphorylation (Mai et al., 2022). Furthermore, DHCR24 overexpression prevented Ras–MEK–ERK overactivation, decreasing tau hyperphosphorylation.

Alternatively, Leucine-rich repeat kinase 2 (LRRK2), a protein prevalent in PD, may play a role in tauopathy development. LRRK2 mutations are among the most common genetic causes of PD, both in familial and sporadic cases. LRRK2 provides an interesting link on small GTPases targeting different neurodegenerative diseases such as AD and PD. Although LRRK2 mutations are not common in AD, it is associated with the downstream kinase substrate Rab subfamily proteins including Rab10 (Liu et al., 2020).

A study analyzing LRRK2-mediated Rab10 phosphorylation in AD patient hippocampal tissues revealed that the pRab10-T73 epitope was associated with NFTs (Yan et al., 2018). Rab10 was similarly found to be highly co-localized with hyperphosphorylated tau. Rab10 was further demonstrated to associate with tau phosphorylation when tauopathy mice treated with LRRK2 inhibitor JZ1.40 exhibited a reversal of Rab10 overactivation and tau hyperphosphorylation (Castro-Sánchez et al., 2020). Besides LRRK2 affecting Rab activity and tau phosphorylation, LRRK2 may achieve similar results through activating MAPK/ERK pathways. Evidence demonstrates that LRRK2 can bind to small GTPases and act as a scaffold to facilitate MAPK/ERK activation (Boon et al., 2014). If LRRK2 mediates tau phosphorylation through the MAPK/ERK pathway, this would corroborate similar instances of small GTPase regulation of tau hyperphosphorylation by MAPK/ERK activity (Lu et al., 1993; Greenberg et al., 1994; Kim et al., 2011; Kirouac et al., 2017; Mai et al., 2022).

Equally important, the Rho GTPase subfamily may regulate tau phosphorylation through means other than APP, MAPK/ERK, and GSK-3 interactions. Rho GTPases can affect tau hyperphosphorylation through direct GAP, GEF, GDI, or effector modulation. For example, Cdc42, a classical Rho subfamily member and crucial regulator of synaptic plasticity, is inactivated by Cdc42GAP. The analysis of a heterozygous Cdc42GAP mouse model with elevated Cdc42 activity suggested that the Cdc42GAP deficiency induced and accelerated AD-like phenotypes while overexpression of dominant-negative Cdc42 reversed synaptic loss and tau hyperphosphorylation (Zhu et al., 2023). The importance of Cdc42 regulation in AD can be further implicated in a recent study in which the modulation of Cdc42 interaction with its selective GEF, intersectin 1 (ITSN1), attenuated AD-like behavior and reduced the hyperphosphorylation of tau at serine 396/404 (or PHF-1) site in the 3xTg-AD mouse model (Malasala et al., 2024).

In another study, RhoGDIα, an inhibitor of Rho activity, was shown to interact with and directly bind to tau (Zhang et al., 2023). Forced expression of RhoGDIα in Aβ25-35- and H/R-induced PC12 cells reduced tau hyperphosphorylation and inhibited Caspase-3 activity. In addition, forced RhoGDIα expression in AD and vascular dementia mouse models ameliorated associated pathological symptoms.

A different study revealed that CDK5 could regulate tau phosphorylation via Rac1. In 24-month-old 3xTg-AD mice, CDK5 dysregulation led to tau hyperphosphorylation, whereas CDK5 KD with RNAi reduced hyperphosphorylated tau and NFTs (Posada-Duque et al., 2015). Along with CDK5 KD, p35 over-expression, and constitutively active Rac1 in primary hippocampal neurons could induce similar neuroprotective effects. These findings appear to be at odds with a study that found a correlation between Rac1 activation and tau hyperphosphorylation in 6-week-old 3xTg-AD mice (Borin et al., 2018). These data suggest that the Rac1 associated manifestation of AD tau pathogenesis may depend on the disease stage, and the different upstream targeting of Rac1 regulation may prove valuable in preventing tau hyperphosphorylation.

Likewise, dysregulation of the downstream effectors of Rho GTPase, for example, Rho kinases (ROCK1 and ROCK2), may play a role in pathways associated with tau hyperphosphorylation. Specifically, studies showed that ROCK1 could phosphorylate the Phosphatase and tensin homolog (PTEN) to promote tau dephosphorylation (Wang et al., 2016). Furthermore, ROCK1 KD by microRNA-146a induced abnormal tau hyperphosphorylation, and supplemental analysis of AD patient brain tissue revealed ROCK colocalization with hyperphosphorylated tau in early NFTs. In addition, Caspase-2, an activator of ROCK2, was required for cognitive decline in human-APP transgenic mice (Pozueta et al., 2013). This was also demonstrated by decreased tau phosphorylation levels in J20 mice with Caspase-2 KO. On the other hand, ROCK2 inhibition by fasudil in mouse hippocampal neurons reduced tau phosphorylation (Gao et al., 2019). These findings suggest that significant ROCK dysregulation, whether regulated by Rho or other proteins, can lead to tau hyperphosphorylation.

While a large number of studies focus on tau phosphorylation, it is critical to consider other post-translational modifications (PTMs) that may contribute to tauopathy (Avila et al., 2004). Similarly to how small GTPases are implicated in tau phosphorylation, small GTPases may play a role in other potentially pathogenic tau PTMs. A reduction in Rhes via lonafarnib coincided with a similar decrease in tau SUMOylation and ubiquitination (Hernandez et al., 2019). Other evidence suggests that Rho and ROCK may activate Caspase 3 in a positive-feedback loop-like manner (Shi and Wei, 2007). Consequently, Rho activity may create tau PTMs downstream, as Caspase 3 demonstrates the ability to generate tau truncations commonly seen in AD brain tissues (Gu et al., 2020). Ras may play a part in its acetylation. According to Alquezar et al. (2021), CREB-binding protein, a histone acetyltransferase, appears to induce tau acetylation and shows altered levels in AD patients and an AD mouse model. Another study demonstrated the ability of the Ras/MAPK pathway to recruit CREB-binding protein (Foulds et al., 2004), suggesting that the Ras small GTPase pathway may potentially trigger tau PTMs through acetylation.

## 5 The roles of small GTPase in tau aggregation

**Figure 4:**
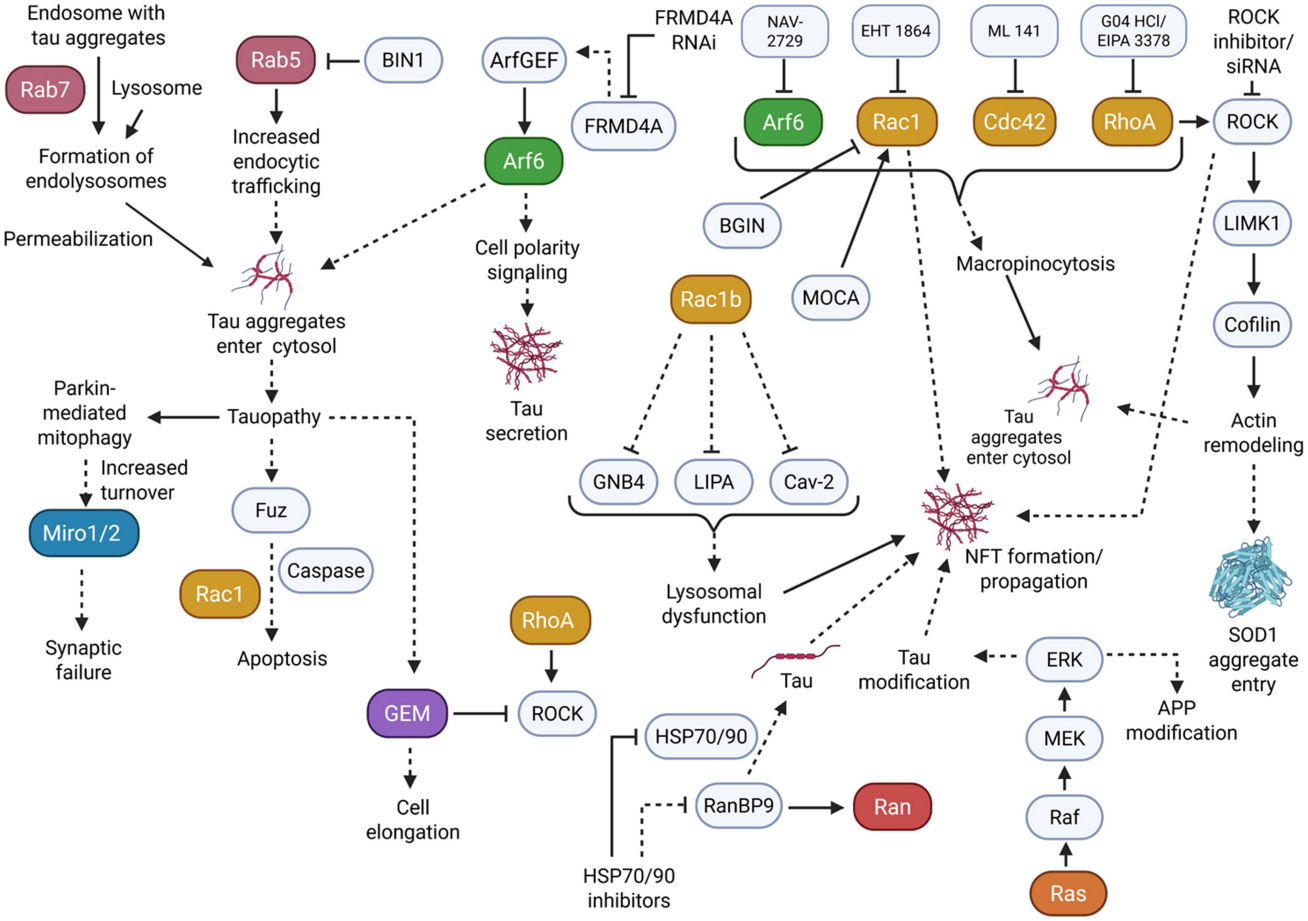
Many small GTPase subfamilies play a role in the propagation of tauopathy. Mainly, dysregulation in vesicular trafficking and membrane dynamics resulting from small GTPase activity can induce an increased entrance/secretion of tau aggregates and the formation of NFTs to further burden cells. Solid arrowheads indicate observed or reported connections that activate/upregulate a protein, signaling molecule, or cellular process; dotted arrowheads indicate unknown or hypothetical/indirect connections that activate/upregulate a protein, signaling molecule, or cellular process. Solid flathead arrows indicate observed or reported connections that inhibit/downregulate a protein, signaling molecule, or cellular process; dotted flathead arrows indicate unknown or hypothetical/indirect connections that inhibit/downregulate a protein, signaling molecule, or cellular process. Curly brackets represent multiple molecules or proteins involved in one of the previously described connections.

In addition to small GTPase activity affecting tau hyperphosphorylation, small GTPases and their associated pathways may further play a part in the propagation and formation of tau aggregates and NFTs (Figure 4).

The Rab small GTPase subfamily, which plays a key role in membrane trafficking, may become dysregulated and promote the subsequent propagation of tauopathy. This dysregulation can be demonstrated in HEK 293T cells and tau transgenic rTg4510 mice. For example, Rab7 overexpression substantially increased tau aggregation through membrane permeabilization of endolysosomes (Polanco et al., 2021). These effects were remediated by Rab7 KD to modify lysosomal function. Studies using rat neurons showed that Rab5 was overactivated due to low BIN1-amphiphysin2 (BIN1), which displayed tau pathology propagation (Calafate et al., 2016). Notably, low BIN1 expression increased the internalization of aggregates, whereas BIN1 overexpression prevented these effects. These findings indicate that excessive Rab activity could promote the beginnings of tau pathology.

As seen with Rab GTPase dysregulation, dysregulation in Arf GTPase can also affect vesicular transport pathways to contribute to elevated tau aggregation. To demonstrate, in another study using HEK293T cells, Arf activity was necessary for tau secretion (Yan et al., 2016). More specifically, tau secretion induced by FRMD4A only occurred in the presence of Arf GEF interactors, while Arf6 and cell polarity signaling stimulated tau secretion. This secretion was effectively blocked by FRMD4A RNAi in mature cortical neurons. On the other hand, in human iPSC (induced pluripotent stem cell) neurons, Arf6 also appears to positively regulate tau aggregate entry by macropinocytosis, as the uptake and propagation of tau aggregates was attenuated by Arf6 inhibition (Krupa et al., 2025).

Further evidence implicates the Rho small GTPase family in tau pathology propagation by affecting the cytoskeletal system. This was seen in a study using the SOD1G93A transgenic mouse model of amyotrophic lateral sclerosis (ALS). Rho GTPase, ROCK1, and LIMK1 kinases activity induced cortical actin remodeling for the cell entry of tau aggregates (Zhong et al., 2018). Particularly, inhibiting Rho and ROCK increased cofilin-1 activity to change actin dynamics and elevate aggregate entry. Rho family members also regulate tau aggregate entry through macropinocytosis, similarly to Arf6, in iPSC neurons. Rac1, Cdc42, and RhoA inhibition were able to reduce the uptake of tau aggregates by macropinocytosis (Krupa et al., 2025).

As opposed to secreting or taking in aggregates, Rho-related tau propagation also appears to occur through inducing the formation of NFTs and aggregates. For example, Rac1b, a constitutively active splice variant of Rac1 GTPase, colocalized significantly with tau in AD human brain tissues (Perez et al., 2012). Further single-cell gene expression profiling showed down-regulation of caveolin 2, GNB4, and lipase A in AD Rac1b-positive/p75^NTR^-labeled cholinergic neurons compared with Rac1b-negative/p75^NTR^-labeled neurons, implicating Rac1b as a modulator of NFT formation and membrane dysfunction in AD.

As previously described, ROCK may also play a role in tau pathology. ROCK inhibitor Y27632 treatment on ischemic rats reduced PHF immunoreactivity, demonstrating a potential for the positive regulation of ROCK on NFT formation (Castro-Alvarez et al., 2011).

Similarly, in another study, Rac1 signaling in HeLa cells was affected by BARGIN (BGIN), a brain-specific RhoGAP variant (Huang et al., 2013). BGIN activity caused membranous Rac1 inactivation, while AD brain tissues revealed a colocalization of BGIN and Rac1 with NFTs. However, studies using APP-expressing cell models have shown that increased levels of Aβ correlate with increased BGIN/poly-Ubiquitination interactions. This suggests that BGIN may be involved in Rac1 inactivation in response to proteotoxicity in AD. Alternatively, the loss of Modifier of cell adhesion (MOCA), a GEF of Rac1 typically found in NFTs, decreases cofilin inactivation and alters LIMK activity (Chen et al., 2009). These changes resulted in axonal degeneration and sensorimotor impairments in mice. Considering the complex roles of Rac1 in modulating tau phosphorylation, the close association of Rac1 and its regulators in NFTs may implicate a regulatory role of Rac1 in NFT formation and propagation.

In addition to Rho GTPases affecting tau aggregation, tauopathy models may have a reciprocal effect on Rho and Rho-related proteins. In particular, Mitochondrial Rho GTPase 1 (Miro1) experienced an increased turnover in tauopathy mouse neurons as a result of an extensive activation of Parkin-mediated mitophagy (Jeong et al., 2022). Overexpressing Miro1 levels rescued mitochondrial anterograde movement, reversing the effects of synaptic failure induced by tau accumulation. In another study, a *Drosophila* model expressing excessive tau induced the upregulation of Fuz, a protein involved in planar cell polarity signaling (Chen et al., 2018). This upregulation triggered neuronal apoptosis through the Rac1 GTPase/Caspase signaling pathways, consequently indicating that Rac1 downstream targeting may also relieve neurodegeneration as a result of tauopathy.

Further evidence demonstrates that Gem GTPase, an extended Ras superfamily member in the RGK subfamily and a negative regulator of the Rho-ROCK pathway, is also affected by tau interactions. Gem was significantly elevated in the brains of tau-deficient mice. Overexpressed Gem induced cell elongation in Chinese hamster ovary (CHO) cells that do not express tau (Oyama et al., 2004). Co-expression of tau led to anti-elongation activity in CHO cells.

Separate evidence suggests that small GTPases other than Rho, Arf, and Rab may be involved in tau aggregation. This is seen in part through a study that demonstrated activating the Ras-dependent MAPK/ERK cascade in AD patient brain samples allowed the mediation of post-transcription modification of APP and tau (Gärtner et al., 1999). Notably, Ras was significantly elevated in the initial stages of AD before SP and NFT formation. These results could potentially implicate the Ras pathway activity as either an initiator of tau propagation or a consequence of other neurodegenerative pathways. Similarly to Ras, Ran-binding protein 9 (RanBP9), a regulator of the Ran GTPase, was found to be highly elevated in AD patient brains and AD mouse models (Woo et al., 2017). Inducing overexpression or KD of RanBP9 caused changes in tau levels, and RanBP9 diminished the anti-tau potency of heat shock protein (HSP70/90) inhibitors.

Literature suggests that the misfolded and aggregated tau can further induce the development of tauopathy via prion-like replication mechanisms. Notably, accelerated misfolding of tau can occur through interactions between misfolded/aggregated tau and native tau, which is further exacerbated through seeding and spread to other cells (Ayers et al., 2018; Jackson et al., 2022). As previously elaborated upon, small GTPases, particularly those of the Rab, Rho, and Arf subfamilies, demonstrated the ability to induce tau aggregation, tau seeding, and secretion. By altering membrane dynamics and vesicular transport pathways, the dysregulation of small GTPases appears to contribute to the prion-like pathogenesis of tau.

## 6 The roles of small GTPase in tau clearance

**Figure 5:**
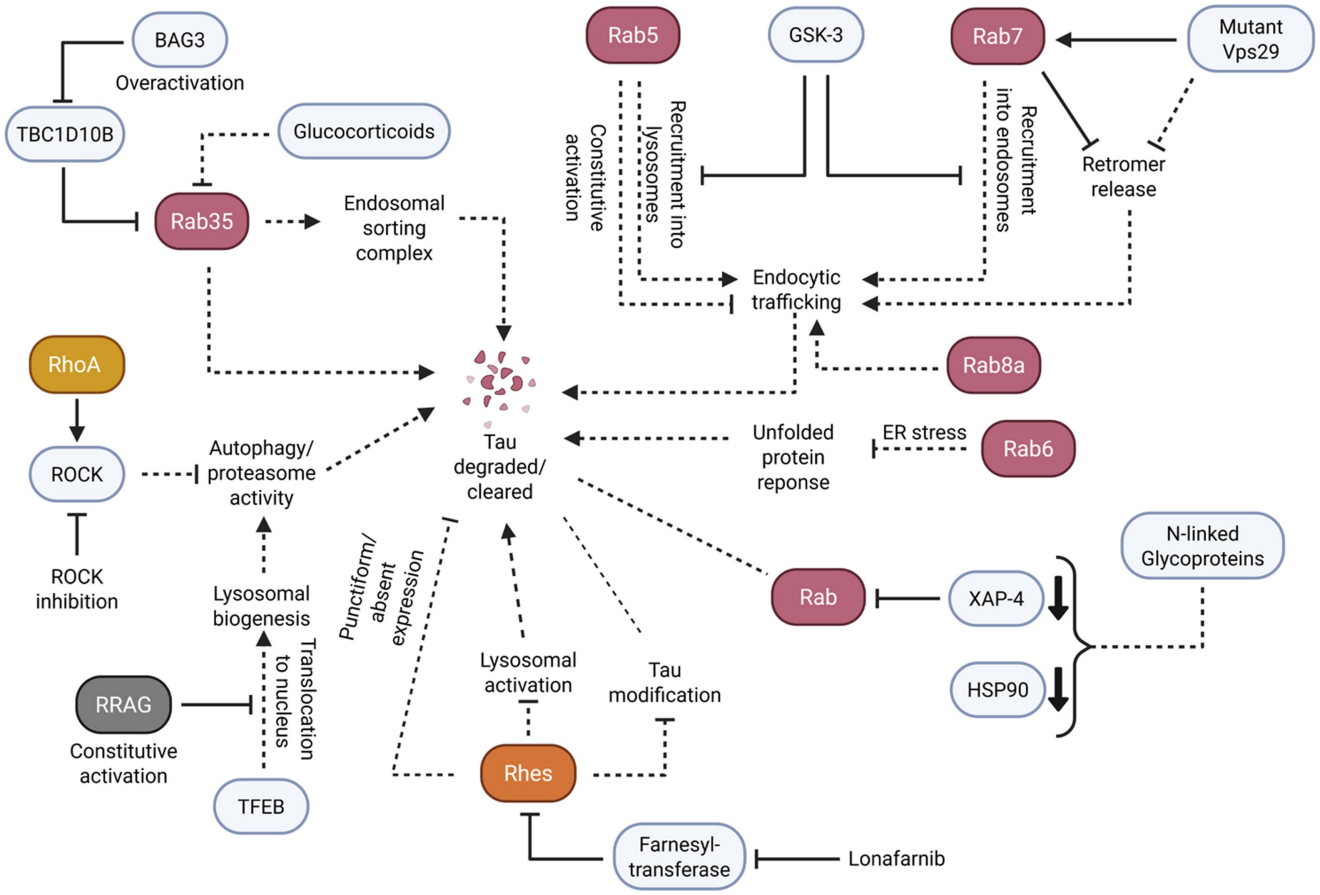
Small GTPases facilitate the proper clearance of tau and tau aggregates through the careful regulation of endosomal/lysosomal trafficking and activation of autophagy responses. Solid arrowheads indicate observed or reported connections that activate/upregulate a protein, signaling molecule, or cellular process; dotted arrowheads indicate unknown or hypothetical/indirect connections that activate/upregulate a protein, signaling molecule, or cellular process. Solid flathead arrows indicate observed or reported connections that inhibit/downregulate a protein, signaling molecule, or cellular process; dotted flathead arrows indicate unknown or hypothetical/indirect connections that inhibit/downregulate a protein, signaling molecule, or cellular process. Dotted lines without arrowheads or flathead arrows represent an unknown or hypothetical/indirect connection between proteins, signaling molecules, and/or cellular processes. Curly brackets represent multiple molecules or proteins involved in one of the previously described connections. Small bold, downturned arrowheads represent a decrease in protein levels.

As well as small GTPases regulating tau aggregation, literature suggests that small GTPases play a role in the clearance of tau aggregates. This clearance is particularly demonstrated through lysosomal pathways. Careful regulation of these small GTPase pathways appears to be critical to clearance, as their dysregulation may promote further tau aggregation (Figure 5).

In particular, the Rab small GTPase subfamily demonstrated potential key roles in tau clearance through lysosomal and endolysosomal processing. Active Rab35 appears to facilitate the proper functioning of the endosomal sorting complex to mitigate tau aggregation. For example, in human AD brains and P301S tau transgenic mice, BAG3 overexpression rescued TBC1D10B-induced Rab35 inactivation (Lin et al., 2022). Conversely, Rab35 inactivation yielded impairment in the downstream endosomal sorting complex, impeding lysosomal fusion. This outcome consequently led to a failure of tau clearance. Likewise, Rab35 inactivation induced by glucocorticoids in N2a cells impaired tau degradation, while restoring Rab35 activity ameliorated the effects of glucocorticoid-induced tau accumulation (Vaz-Silva et al., 2018).

Like Rab35, Rab5 and Rab7 regulate endosomal function, particularly in early and late endosomes, which is crucial to cellular clearance. To illustrate these functions, GSK-3 inhibition in MEF cells increased the recruitment of Rab5 into endosomes and the clustering of Rab7/RILP into lysosomes (Avrahami et al., 2020). Further results revealed that constitutively active Rab5 caused the dysfunction of endocytic traffic despite GSK-3 inhibition, preventing a restoration of lysosomal acidification. Alternatively, in *Drosophila* neurons, Rab7 facilitated the endosomal binding and release of retromer, a protein-recycling complex part of the endolysosomal pathway (Ye et al., 2020). Prolonged Rab7 activation and retromer binding caused by a mutant retromer Vps29 subunit led to lysosomal stress and impaired clearance. In addition, reducing the amount of Rab7 rescued clearance by autophagy in Vps29 mutant brains. As a result, Rab5 and Rab7 regulation of endocytic traffic appears to be crucial to clearance pathways, supporting its potential role in tau clearance.

Rab8a, another protein involved in protein transport, appears to have a comparable role in aggregate clearance. For example, one study that used H4 cells with induced Rab8a overexpression resulted in a reduction in total tau and phosphorylated tau (Parmar, 2024). In comparison, with mutant, non-functioning Rab8a, no reduction in tau was observed. It is important to note that Rab8a has already been demonstrated to accelerate endocytosed Aβ trafficking to lysosome expression, suggesting that Rab8a can also regulate tau clearance through similar means.

Outside of the Rab subfamily, the Rho and RRAG GTPases may also regulate tau clearance pathways through lysosomes and autophagy. For instance, one study that inhibited ROCK in tau-expressing M1C cells and primary cultured mouse neurons reduced the total and phosphorylated tau protein (Hamano et al., 2020). In addition, ROCK inhibition activated the autophagy and proteasome pathways for the degradation of tau protein. Another study that used cultured primary mouse neurons demonstrated that the nuclear translocation of transcription factor EB (TFEB) mediated lysosomal biogenesis and autophagy to clear aggregated tau (Akwa et al., 2023). On the contrary, neurons with constitutively active RRAG GTPases sequestered TFEB in the cytosol, preventing tau clearance.

In addition to small GTPases managing tau clearance through lysosomal/endosomal pathways, small GTPases may facilitate tau clearance through post-translational control and modification pathways. An example of this is seen in a study examining the brains of AD and control patients. Analysis revealed that there was Rab6 upregulation in the AD temporal cortex during the same stage as unfolded protein response (UPR) activation (Scheper et al., 2007). Notably, there was some colocalization of Rab6 and hyperphosphorylated tau, and there was a strong correlation between Rab6 and BiP, a protein involved in protein processing. Additional evidence supports this association between Rab6 and the UPR, as Rab6 activity was shown to modulate the UPR after prolonged ER stress (Elfrink et al., 2012). These studies suggest that Rab6 may play a role in redirecting proteins, including tau, to the ER for subsequent degradation.

In a separate study, AD brains exhibited altered levels of concanavalin-A (Con-A), a protein involved in N-linked-glycoprotein binding (Owen et al., 2009). AD hippocampus contained decreased Con-A-associated protein levels of Rab GDI XAP-4 and HSP90, which are notably involved in regulating membrane trafficking and tau association. The data of this study may further support Rab dysregulation as a contributor of tau clearance failure.

Similarly, Rhes, a Ras subfamily-related small GTPase, appears to manage tau clearance with post-translational control and modification pathways. To demonstrate, inhibiting farnesyltransferase with lonafarnib in a mouse model of tauopathy exhibited a reduction in Rhes and a decrease in brain atrophy, tau inclusions, tau sumoylation, and tau ubiquitination (Hernandez et al., 2019). Furthermore, the reduction of tau pathology through Rhes inactivation was operated through lysosomal activation. In another study, histological examination of human tauopathy brains revealed that neurons with punctiform or absent patterns of Rhes tended to include elevated levels of tau aggregates while neurons with diffuse Rhes distribution had lower tau (Ehrenberg et al., 2021). Likewise, these Rhes distributions also affected the frequency of post-translational tau modifications.

Here, a summary table is presented to illustrate the functional relationship between small GTPases and tau protein posttranslational phosphorylation and distribution, aggregation and propagation, and clearance (Table 1).

**Table 1:** List of small GTPases involved in tau pathogenesis.

Small GTPase subfamilies	Small GTPases	Dysregulation mechanism	Disease process	References
Ras	Ras	Raf/MEK/MAPK; GSK-3	Tau hyperphosphorylation	Lu et al. (1993); Greenberg et al. (1994); Kim et al. (2011); Kirouac et al. (2017); Mai et al. (2022)
	Rhes	Farnesylation	Tau hyperphosphorylation; sumolation, ubiquitination; Clearance	Ehrenberg et al. (2021); Hernandez et al. (2019)
Rho	RhoA	Effector ROCK; RhoGDIα; Macropinocytosis	Tau hyperphosphorylation; Tau aggregation	Krupa et al. (2025); Sayas et al. (2002); Zhong et al. (2018)
	Rac1	CDK5; BGIN; macropincytosis; reciprocal effects downstream of tau accumulation	Tau hyperphosphorylation; Tau aggregation	Borin et al. (2018); Huang et al. (2013); Krupa et al. (2025); Perez et al. (2012); Posada-Duque et al. (2015)
	Cdc42	GAP; GEF; macropinocytosis	Tau hyperphosphorylation; Tau aggregation	Krupa et al. (2025); Malasala et al. (2024); Zhu et al. (2023)
Rab	Rab5	BIN1	Tau aggregation; clearance	Avrahami et al. (2020); Calafate et al. (2016); Pensalfini et al. (2020)
	Rab7	Lysosomal membrane;	Tau aggregation; clearance	Avrahami et al. (2020); Polanco et al. (2021); Ye et al. (2020)
	Rab 8a	Endolysosomal imbalance	Tau hyperphosphorylation; clearance	Parmar (2024)
	Rab 6	BiP and UPR	Tau aggregation; clearance	Elfrink et al. (2012); Scheper et al. (2007)
	Rab 35	Endolysosomal imbalance	Tau clearance	Lin et al. (2022); Vaz-Silva et al. (2018)
	Rab unspecified	Rab GDI XAP-4 and HSP90	Tau clearance	Owen et al. (2009)
	Rab 10	LRRK2	Tau hyperphosphorylation	Castro-Sánchez et al. (2020); Yan et al. (2018)
Arf	Arf6	GEF; macropinocystosis	Tau aggregation; tau secretion	Krupa et al. (2025); Yan et al. (2016)
Ran	Ran	RanBP9	Tau expression	Woo et al. (2017)
Miro	Miro1	Reciprocal effects downstream of tau accumulation	Tau aggregation	Jeong et al. (2022)
RRAG	RRAG unspecified	TFEB; Lysosomal biogenesis/ autophagy	Tau clearance	Akwa et al. (2023)
RGK	Gem	Rho-ROCK inhibition	Tau interactions	Oyama et al. (2004)

## 7 Targeting small GTPases as a treatment option to clear tau pathologies

The important modulatory roles of small GTPases in AD tau pathogenesis, such as hyperphosphorylation, aggregation, and propagation, suggest that this large Ras superfamily of signaling proteins may be a therapeutic target for AD and ADRD. They are particularly suitable for drug development in chronic diseases like neurodegenerative disorders because they are molecular switches, and their activation and inactivation can be controlled by GEFs, GAPs and GDIs to achieve tissue and cell selectivity.

Over the past decades, many small GTPase modulators have been developed. They are being applied widely in investigations of cardiovascular disorders and stroke, stem cell functions, infectious and inflammatory diseases, neurological disorders, and cancer. However, there are very few clinical applications of small GTPase modulators for neurodegenerative diseases (Table 2).

**Table 2:** List of molecules and proteins targeting small GTPases affecting tau pathology.

Small GTPase subfamilies	Small GTPases	Therapeutic molecule/protein	Therapeutic effects	References
Ras	Ras	DHCR24	Decreased Ras/MAPK activation, decreased tau hyperphosphorylation	Mai et al. (2022)
	Rhes	Lonafarnib	Lysosomal activation decreases brain atrophy, tau inclusions, and tau sumoylation/tau ubiquitination	Hernandez et al. (2019)
Rho	RhoA	C3 transferase	Reduction in tau hyperphosphorylation	Sayas et al. (2002)
		Rhosin hydrochloride (5003); Ethylisopropyl amiloride (EIPA; 3,378)	Discontinuation of tau aggregate uptake/spreading by macropinocytosis	Krupa et al. (2025)
	Rac1	EHT 1864 (E1657)	Discontinuation of tau aggregate uptake/spreading by macropinocytosis	Krupa et al. (2025)
		BGIN	Unclear; Membranous Rac1 inactivation and colocalization with Rac1 and NFTs	Huang et al. (2013)
	Cdc42	ML 141 (SML0407)	Discontinuation of tau aggregate uptake/spreading by macropinocytosis	Krupa et al. (2025)
		ZCL279	Modification of AD-like behavior in the 3xTg-AD mouse model; Reduction in tau phosphorylation at 396/404 (PHF1) sites	Malasala et al. (2024)
Rab	Rab5	BIN1-amphysin2	Negative regulation of tau pathology propagation	Calafate et al. (2016)
	Rab 35	BAG3	Enhanced amelioration of Rab35 activation rescues lysosomal fusion and clearance of tau aggregates	Lin et al. (2022)
	Rab 10	JZ1.40	Amelioration in Rab10 overactivation, decreased tau phosphorylation	Castro-Sánchez et al. (2020)
Arf	Arf6	NAV-2729 (SML2238)	Discontinuation of tau aggregate uptake/spreading by macropinocytosis	Krupa et al. (2025)

Targeting the RhoA downstream effector ROCK is among the most notable therapeutic approaches reviewed in neurodegenerative disease research (Zheng et al., 2025; Weber and Herskowitz, 2021). Fasudil and its derivatives were approved in the 1990’s for clinical treatment of vasospasm. But they have also inhibited ROCK and decreased phosphorylated tau in AD neuro-spheroids (Giunti et al., 2023) and in primary neurons (Hamano et al., 2020). They are currently in clinical trials for tauopathies of progressive supranuclear Palsy-Richardson syndrome and corticobasal syndrome in the US (NCT04734379). Fasudil is also under compassionate use for amyotrophic lateral sclerosis in the US (NCT03792490) and Europe (2017-003676-31) (Zheng et al., 2025).

Evidence also shows that other classical Rho GTPases, such as Rac1 and Cdc42, are dysregulated in AD. Like RhoA signaling, there were conflicting studies about the overactivation or inactivation in Rac1 and Cdc42 associated with AD (Huesa et al., 2010; Nik Akhtar and Lu, 2023; Nik Akhtar et al., 2024). Nevertheless, NSC23766, a Rac1 selective inhibitor that interferes with the interactions of Rac1 and its GEF Tiam, decreased Aβ levels and prevented Aβ42 peptide-induced cell death (Aguilar et al., 2017). ZCL279, which interferes with the interactions of Cdc42 with its selective GEF, ITSN1, modified AD-like behavior in the 3xTg-AD mouse model and reduced tau phosphorylation at 396/404 (PHF1) sites (Malasala et al., 2024).

Several other small GTPase modulators described here may suggest their targeting potential in AD. Lonafarnib is the first FDA-approved drug for Hutchinson-Gilford progeria syndrome and some types of progeroid laminopathies. It inhibits farnesyltransferase, an enzyme involved in the farnesylation of some small GTPases. The reduction of Ras family protein Rhes and the decreased tau pathologies following lonafarnib treatment (Hernandez et al., 2019) raised the possibility of potential benefits of inhibition of various farnesylated small GTPases in AD.

Finally, LRRK2 inhibitor JZ1.40 reduced Rab10 overactivation and tau hyperphosphorylation in tauopathy mice (Castro-Sánchez et al., 2020). Currently, there are multiple ongoing clinical trials utilizing LRRK2 inhibitors, like BIIB122, DNL201, and DNL151 (Jennings et al., 2023; Hyderi et al., 2025). It remains to be seen whether these LRRK2 inhibitors inhibit PD through Rab subfamily small GTPases and whether the same strategy can apply to AD treatment.

## 8 Summary

Literature survey revealed many small GTPases involved in AD tau pathogenesis. This included all subfamilies in the Ras superfamily of small GTPases, namely, Ras, Ran, Rho, Rab, Arf, and Miro.

Specifically, small GTPases of the different subfamilies can regulate the different phases of AD tau pathogenesis from hyperphosphorylation, to aggregation and propagation. These findings support targeting small GTPases as a potential AD therapeutic strategy.

It is also important to note that for some small GTPases, such as Rac1, evidence demonstrates that both stimulation and inhibition can lead to the attenuation of some aspects of AD pathogenesis. The context-dependent functions of small GTPases suggest that a minimum level of their activity may be important in physiological functions (Socodato et al., 2023). Alternatively, different small GTPases may exert their effects at different stages of AD pathogenesis in a cell- and region-selective manner.
